# *Toxoplasma gondii* bradyzoite-specific BAG1 is nonessential for cyst formation due to compensation by other heat-shock proteins

**DOI:** 10.1186/s13071-024-06339-w

**Published:** 2024-07-30

**Authors:** Weiling Wu, Qiqi Chen, Weihao Zou, Jiating Chen, Di Zhu, Huijing Yang, Lishan Ouyang, Xiaojun Liu, Hongjuan Peng

**Affiliations:** 1 Department of Anesthesiology, The Key Laboratory of Precision Anesthesia & Perioperative Organ Protection, Baiyun Branch, Nanfang Hospital, Nanfang Hospital, Southern Medical University, Guangzhou, Guangdong 510515 People’s Republic of China; 2 Department of Pathogen Biology, Guangdong Provincial Key Laboratory of Tropical Diseases Research, School of Public Health, Ministry of Education, Key Laboratory of Infectious Diseases Research in South China (Southern Medical University), 1023-1063 South Shatai Rd, Guangzhou, Guangdong People’s Republic of China 510515

**Keywords:** *Toxoplasma gondii*, *bag1*, CRISPR/Cas9, Heat-shock protein, Tachyzoite and bradyzoite transformation

## Abstract

**Background:**

*Toxoplasma gondii* is an opportunistic pathogenic protozoan that infects all warm-blooded animals, including humans, and causes zoonotic toxoplasmosis. The bradyzoite antigen 1 (BAG1), known as heat-shock protein (HSP)30, is a specific antigen expressed during the early stage of *T. gondii* tachyzoite–bradyzoite conversion.

**Methods:**

A *bag1* gene knockout strain based on the *T. gondii* type II ME49 was constructed and designated as ME49Δ*bag1*. The invasion, proliferation, and cyst formation efficiency in the cell model and survival in the mouse model were compared between the ME49 and ME49Δ*bag1* strains after infection. Quantitative polymerase chain reaction (qPCR) was used to detect the transcriptional level of important genes, and western-blot was used to detect protein levels.

**Results:**

ME49Δ*bag1* displayed significantly inhibited cyst formation, although it was not completely blocked. During early differentiation induced by alkaline and starvation conditions in vitro, the proliferation of ME49Δ*bag1* was significantly accelerated relative to the ME49 strain. Meanwhile, the transcription of the HSP family and bradyzoite formation deficient 1 (*bfd1*) were significantly enhanced. The observed upregulation suggests a compensatory mechanism to counterbalance the impaired stress responses of *T. gondii* following *bag1* knockout. On the other hand, the elevated transcription levels of several HSP family members, including HSP20, HSP21, HSP40, HSP60, HSP70, and HSP90, along with BFD1, implied the involvement of alternative regulatory factors in bradyzoite differentiation aside from BAG1.

**Conclusions:**

The data suggested that when *bag1* was absent, the stress response of *T. gondii* was partially compensated by increased levels of other HSPs, resulting in the formation of fewer cysts. This highlighted a complex regulatory network beyond BAG1 influencing the parasite’s transformation into bradyzoites, emphasizing the vital compensatory function of HSPs in the *T. gondii* life cycle adaptation.

**Graphical Abstract:**

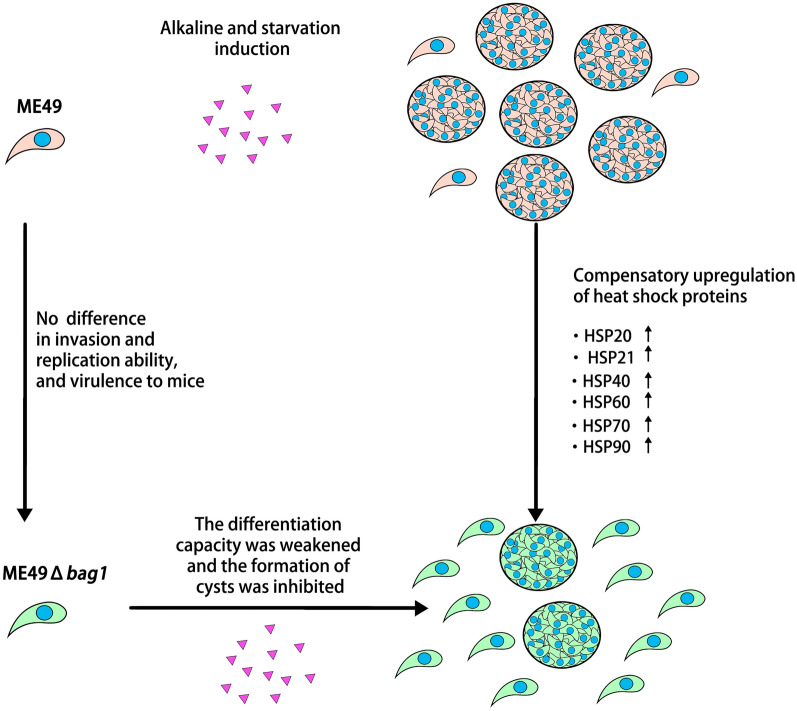

## Background

*Toxoplasma gondii* is an opportunistic, obligate intracellular protozoan that causes zoonotic toxoplasmosis. This parasite has a worldwide distribution and poses a significant health risk to populations, with about one-third of the world’s population showing seropositivity against the parasite [1, 2]. In immunocompromised populations, *T. gondii* infection can cause encephalitis, retinochoroiditis, and damage to various organs [3]. For pregnant women who become infected for the first time with *T. gondii* during pregnancy, infection can lead to miscarriage, premature delivery, teratogenic effects, or stillbirth, and infected fetuses may develop symptoms such as hydrocephalus, brain calcifications, retinochoroiditis, and mental and movement disorders [4]. Throughout the developmental stages of *T. gondii* infection, the interconversion between rapidly dividing tachyzoites in the acute phase and slowly dividing bradyzoites in the chronic phase is crucial and is the main target for toxoplasmosis treatment. Inhibiting this transformation process is a prominent research focus and a critical control point for the treatment of toxoplasmosis [5].

*Toxoplasma gondii* ME49 strain, a type II, cyst-forming strain, exhibits a higher rate of spontaneous cyst formation in culture than virulent strains such as the RH strain, and has been reported to be most frequently associated with human disease-related type [6]. *Toxoplasma gondii* bradyzoite antigen 1 (BAG1), one of the specific antigens of the bradyzoite stage, is localized in the bradyzoite cytoplasm [7]. It is expressed in the early stage of tachyzoite transformation into bradyzoite, and is detectable for the first time 2–3 days after the initiation of bradyzoite differentiation, which makes it a viable marker for monitoring *T. gondii* disease progression [8, 9].

The *T. gondii* BAG1 protein, also known as heat-shock protein (HSP)30, belongs to the small heat-shock protein (sHSP) family and plays a crucial role in stress response. Knocking down the sHSPs, such as HSP26 in *Saccharomyces cerevisiae *[10] and HSP32 in *Dictyostelium discoideum *[11], significantly disrupts the differentiation processes in these organisms. These sHSPs also play a role in regulating apoptosis and differentiation [12]. A growing body of evidence indicates that the differentiation events of bradyzoites are mediated by stress and potentially utilize the same pathways as stress-triggered differentiation events in other eukaryotes.

Regarding the entire HSP family in *T. gondii*, HSPs play a significant role in the life cycle of the parasite and in its response to stressful environments, as well as in the interconversion between tachyzoites and bradyzoite. HSP40 is reported to be involved in the molecular chaperone mechanism, which can prevent protein aggregation and induce refolding, repairing stress-damaged proteins; it may play a significant role in the development and survival of *T. gondii* bradyzoites within the hosts [13]. HSP70 is potentially related to the differentiation of *T. gondii* bradyzoites, mainly expressed during the transition from tachyzoites to bradyzoites, playing a vital role in the adaptation of *T. gondii* [14–16]. HSP70 is a potential virulence factor of *T. gondii*, regulating host immune responses during infection of host cells, thereby protecting the parasite from the host’s immunity [17–19]. HSP90 aids in the development of bradyzoites and the invasion and replication of *T. gondii* in host cells [20]. Moreover, it has been shown that knocking out the HSP90 gene in *Toxoplasma *[20], or binding and disrupting HSP90 function with geldanamycin [21], will block the interconversion of tachyzoites and bradyzoites [21].

In this study, clustered regularly interspaced short palindromic repeats (CRISPR)/CRISPR-associated protein 9 (Cas9) technology was utilized to establish a *bag1* gene knockout strain derived from *T. gondii* ME49. The phenotypes of this knockout strain (ME49Δ*bag1*) were analyzed to explore the role of *bag1* in parasite differentiation in vitro and in vivo. This is helpful for understanding the mechanism of *T. gondii* tachyzoite–bradyzoite interconversion, and finding new strategies for the prevention and treatment of toxoplasmosis.

## Methods

### Parasites and cell lines

The ME49Δ*ku80* strain derived from the *T. gondii* type II strain ME49 was propagated in human foreskin fibroblast (HFF) cells (purchased from the American Type Culture Collection, ATCC), which were cultured in Dulbecco’s modified Eagle’s medium (DMEM, Gibco, USA) complete medium supplemented with 10% fetal bovine serum (FBS, Gibco, USA) and 1% penicillin–streptomycin (Thermo Fisher Scientific, USA).

*Toxoplasma gondii* was cultured in DMEM medium supplemented with 1% FBS and 1% penicillin–streptomycin (D1). ME49Δ*bag1* was cultured in D1 supplemented with an additional 3 μM pyrimethamine. All cell and parasite culture conditions were kept at 37 °C with 5% CO^2^.

When a large number of tachyzoites were about to rupture from the HFF cells after infection, the cells were scraped and resuspended in DMEM. The resuspended material was passed three times through a 27-gauge needle attached to a 10-ml syringe to ensure that the host cells were lysed. The lysed tachyzoites were then added to fresh HFF cells for further culturing. The medium used for in vitro transformation contained 0.8% FBS, and 1% penicillin–streptomycin, as well as 20 μmol/l HEPES (pH 8.2 or 9.0).

### Construction of the* bag1* knockout strain ME49Δ*bag1*

Specific single-guide RNA (sgRNA) was designed using the E-CRISPR database (www.e-crisp.org). The ROP18II-Cas9-sgRNA plasmid stored in our laboratory was used as a template [22].

The Q5 site-directed mutagenesis kit (New England Biolabs [NEB]) was used to construct the CRISPR/Cas9 plasmid targeting the 5′-CDS of the *bag1* gene (BAG1-Cas9-sgRNA). The upstream and downstream homology arms of the *bag1* gene (*bag1*-5′UTR, *bag1*-3′UTR) were amplified and tagged with the pyrimethamine resistance marker dihydrofolate reductase (DHFR) by homologous recombination to obtain homologous template fragments.

The *bag1*-Cas9-sgRNA (8 μg) and homologous template fragment (1.5 μg) were transfected into 1 × 10^7^ ME49Δ*ku80* tachyzoites by electroporation [23]. The transformants were then transferred to HFF cells in DMEM containing 1% FBS. *Toxoplasma gondii* was continuously screened for pyrimethamine resistance over three passages, and positive clones were isolated by the limiting dilution method. Tachyzoites were inoculated into HFF cells in 96-well plates at two tachyzoites per well. Strains that were positive for polymerase chain reaction (PCR)1 and PCR2, and negative for PCR3, were amplified and cultured to obtain monoclonal knockout strains.

### Detection of SAG1 or BAG1 by western blot

Parasites and cells were lysed with radioimmunoprecipitation assay (RIPA) buffer (Beyotime, China) containing 1% phenylmethylsulfonyl fluoride (PMSF) on ice, centrifuged at 13,000×*g* for 5 min at 4 °C. The supernatant was separated to obtain total proteins, and 50 μl of supernatant was taken out and mixed with 250 μl of 6× protein-loading buffer in an Eppendorf tube. This tube was placed in a metal bath at 100 °C for 10 min and in ice water. The boiled samples were subjected to sodium dodecyl sulfate–polyacrylamide gel electrophoresis (SDS-PAGE). The proteins in the gel were transferred to the polyvinylidene difluoride (PVDF) membrane. The PVDF membrane was blocked with 5% bovine serum albumin (BSA) at 37 °C for 1 h. Mouse monoclonal antibody against *T*. *gondii* BAG1 and rabbit polyclonal antibody against *T*. *gondii* surface antigen 1 (SAG1) were prepared in our laboratory and diluted 1:200 with 5% BSA blocking buffer. The PVDF membrane was incubated in the anti-SAG1 or anti-BAG1 antibody overnight at 4 °C. The next day, the membranes were washed with TBST three times for 5 min each. Horseradish peroxidase (HRP)-goat anti-rabbit or HRP-goat anti-mouse antibody diluted 1:6000 with blocking buffer was added to the membranes and incubated for 1 h at 37 °C. After TBST washing, color development was performed using a Bio-Rad ECL luminescent solution kit (Bio-Rad).

### Plaque assay for ***T. gondii*** multiplication

HFF cells in 12-well plates with 100% confluence were infected with 1000 tachyzoites of ME49 and ME49Δ*bag1* per well. At 4 h after infection, the uninvaded parasites were aspirated with the culture medium, and the cells were then cultured in fresh DMEM complete medium for 7 days. After washing three times with PBS, the cells were fixed in 1 ml of 4% paraformaldehyde for 10 min. After again washing three times with PBS, the cells were stained in 1% crystal violet dye for 30 min. The plaques were then rinsed with tap water and air-dried. The area of each plaque was calculated under a microscope.

### Detection of *T. gondii* invasion efficiency

HFF cells were grown on coverslips in 12-well plates to 100% confluence, and then infected with ME49 or ME49Δ*bag1* tachyzoites for 1 h at a multiplication of infection (MOI) of 3. Uninvaded tachyzoites were aspirated along with the culture medium and the cells were washed three times with PBS. The cells were then fixed in 4% polyformaldehyde for 10 min, rinsed three times with PBS, and blocked in 10% BSA blocking buffer for 2 h at 37 °C. The blocking buffer was aspirated and a rabbit polyclonal antibody against *T. gondii* (ab138698, Abcam) at 1:500 dilution in blocking buffer was added to each well and incubated overnight at 4 °C. The next day, the primary antibody was aspirated and the wells were rinsed three times with PBS. The cells were then incubated with Alexa Fluor 594 goat anti-rabbit (1:500) for 2 h. After discarding the fluorescence secondary antibody, the cells were washed three times with PBS, permeabilized with 0.5% Triton X-100 diluted in PBS for 10 min at room temperature, and washed three times again with PBS. Following the blocking for 2 h, overnight incubation with the rabbit polyclonal antibody against *T. gondii*, and washing three times with PBS, the cells were incubated with Alexa Fluor 488 goat anti-rabbit (1:500) for 2 h the next day. After the secondary antibody was aspirated, the cells were washed three times with PBS. The coverslips were taken out and rinsed three times with double-distilled water (ddH_2_O), air-dried, and mounted with DAPI Fluoromount-G (SouthernBiotech). The number of intracellular and extracellular *T. gondii* tachyzoites per 100 host cells was determined under a fluorescence microscope, and the invasion rate of *T. gondii* was calculated as 100% × (total *T. gondii* − extracellular *T. gondii*)/total *T. gondii*.

### Proliferation assay for *T. gondii*

HFF cells were grown on coverslips in 12-well plates to 100% confluence, and then infected with ME49 or ME49Δ*bag1* tachyzoites for 4 h at an MOI of 1. Uninvaded tachyzoites were aspirated along with the culture medium, and the cells were washed three times with PBS. The cells were then fixed in 4% polyformaldehyde for 10 min. After washing three times with PBS, the cells were permeabilized with 0.5% Triton X-100 diluted in PBS for 10 min at room temperature and again washed three times with PBS. Following the blocking for 2 h, overnight incubation with the rabbit polyclonal antibody against *T. gondii*, and washing three times with PBS, the cells were incubated with Alexa Fluor 488 goat anti-rabbit (1:500) for 2 h the next day. After the secondary antibody was aspirated, the cells were washed three times with PBS. The coverslips were taken out and rinsed three times with ddH_2_O, air-dried, and mounted with DAPI Fluoromount-G (SouthernBiotech). The number of intracellular tachyzoites per 200 parasitophorous vacuoles (PVs) was determined under a fluorescence microscope, and the number of the tachyzoites per PV was compared between ME49 and ME49Δ*bag1* tachyzoites.

### ***Toxoplasma gondii*** bradyzoite transformation assay in vitro

HFF cells were grown in 6-well and 12-well plates on coverslips to 100% confluence and infected with ME49 or ME49Δ*bag1* tachyzoites for 4 h at an MOI of 0.2. Uninvaded tachyzoites were aspirated along with the culture medium and the cells were washed three times with PBS. The parasites and HFF cells were then continuously cultured in D1 at pH 7.2 or DMEM containing 0.8% FBS at pH 8.2 for 24 h, followed by an increase in the pH value to 9.0 and then continuously cultured. The alkaline medium was changed daily to maintain the pH at 9.0. The alkaline stress and starvation condition promoted the transformation of tachyzoites into bradyzoites in vitro. Parasites and host cells in 6-well plates were collected at 36 h intervals for the absolute quantification of the *B1* gene by Quantitative polymerase chain reaction (qPCR), and the growth and proliferation curves of the parasites were plotted to assess in vitro transformation [23]. The two-tailed Student *t*-test was used to compare the *B1* gene of ME49 and ME49Δ*bag1* in the pH 9.0 group after 120 h of culture. In parallel, the total RNA of basal-induced parasites and cells was collected every 24 h using the TRIzol method, and after 96 h of induction, the alkaline medium was exchanged with D1 medium. The total RNA of parasites and cells was prepared every 12 h, and reverse transcription qPCR was performed. The messenger RNA (mRNA) levels of *sag1*, *cst1*, *ldh1*, *ldh2*, *hsp20, hsp21, hsp40, hsp60, hsp70, hsp90, *and *bfd1* genes of *T. gondii* were detected by relative quantification with *gapdh* as the internal control. The transcription curves of these specific genes indicative of parasite transformation in vitro were generated.

### Comparison of ME49 and ME49Δ*bag1* virulence in mouse model

Female specific-pathogen-free (SPF) BALB/c mice aged 6–8 weeks were selected. Before the experiment, the mice were acclimatized under identical conditions for 1 week to reduce stress response. ME49 or ME49Δ*bag1* strains were intraperitoneally injected at a dose of 100 tachyzoites per mouse, with 10 mice in each group. Mice were euthanized at 60 days post-infection (dpi).

### Survival of the two groups of mice was recorded daily for 60 days

*Mouse serum cytokine detection.* Before the mice were euthanized, blood was collected for serum isolation, and subjected to tumor necrosis factor alpha (TNF-α), interferon gamma (IFN-γ), and interleukin (IL)-12 detection using enzyme-linked immunosorbent assay (ELISA) kits (Liankebio).

*Brain parasitic load detection.* The intact brains were excised and homogenized in 2 ml PBS, and 100 μl brain homogenate was used for DNA extraction for *B1* gene detection by qPCR.

*Brain cyst counting.* Brain homogenate (500 μl) was taken out and centrifuged at 1000 g for 5 min, the supernatant was carefully discard, and 1 ml of 10% BSA (diluted in PBS) was added and incubated at room temperature for 2 h. Then, 500 μl monoclonal antibody against BAG1 (diluted 1:200 in 10% BSA) was added to the brain homogenate and incubated overnight at 4 °C. After centrifugation and discarding the supernatant, the pellet was resuspended in 1 ml 10% BSA, and this step 3 was repeated three times. Next, 500 μl of goat anti-mouse immunoglobulin G (IgG) (H+L) Alexa Fluor™ Plus 594 (Thermo Fisher) diluted 1:800 and 1:200 with fluorescein-labeled *Dolichos biflorus* agglutinin (DBA; vector) was added to the brain homogenate pellet, incubated at 37 °C for 2 h, and then resuspended in 250 μl of 10% BSA. Next, 12.5 μl of brain homogenate was smeared on the slides (three slides per mouse) and observed under a fluorescence microscope. The brain cysts on the slide were counted and the mean number of cysts was calculated. The number of cysts per mouse was calculated using the formula of average cyst count × 80.

*Detection of the parasitic load in peritoneal fluid of mice during acute infection.* Another group of female SPF BALB/c mice aged 6–8 weeks were raised under the same conditions. ME49 or ME49Δ*bag1* strains were injected intraperitoneally at a dose of 1000 tachyzoites per mouse to establish an acute infection model. The peritoneal fluid was collected at 9 dpi, after which 5 ml of PBS was injected into the peritoneal cavity of the mice. Genome DNA was extracted using the ALFA-SEQ Tissue DNA kit (FINDROP) and subjected to qPCR for *B1* gene detection.

### Statistical evaluation

The evaluation of statistical data was conducted using SPSS version 20 software (IBM Corp., Armonk, NY, USA). For the assessment of differences between two distinct groups, an unpaired, two-tailed Student *t*-test was employed. In scenarios involving more than two groups, analyses were performed through either one-way analysis of variance (ANOVA) or the Kruskal–Wallis test, depending on the distribution of the data. Survival rates of the mice were analyzed with the log-rank (Mantel–Cox) test to determine their statistical significance. A *P*-value of less than 0.05 was considered statistical significance.

## Results

### Construction of *bag1* knockout *T. gondii* strain ME49Δ*bag1*

**Fig. 1:**
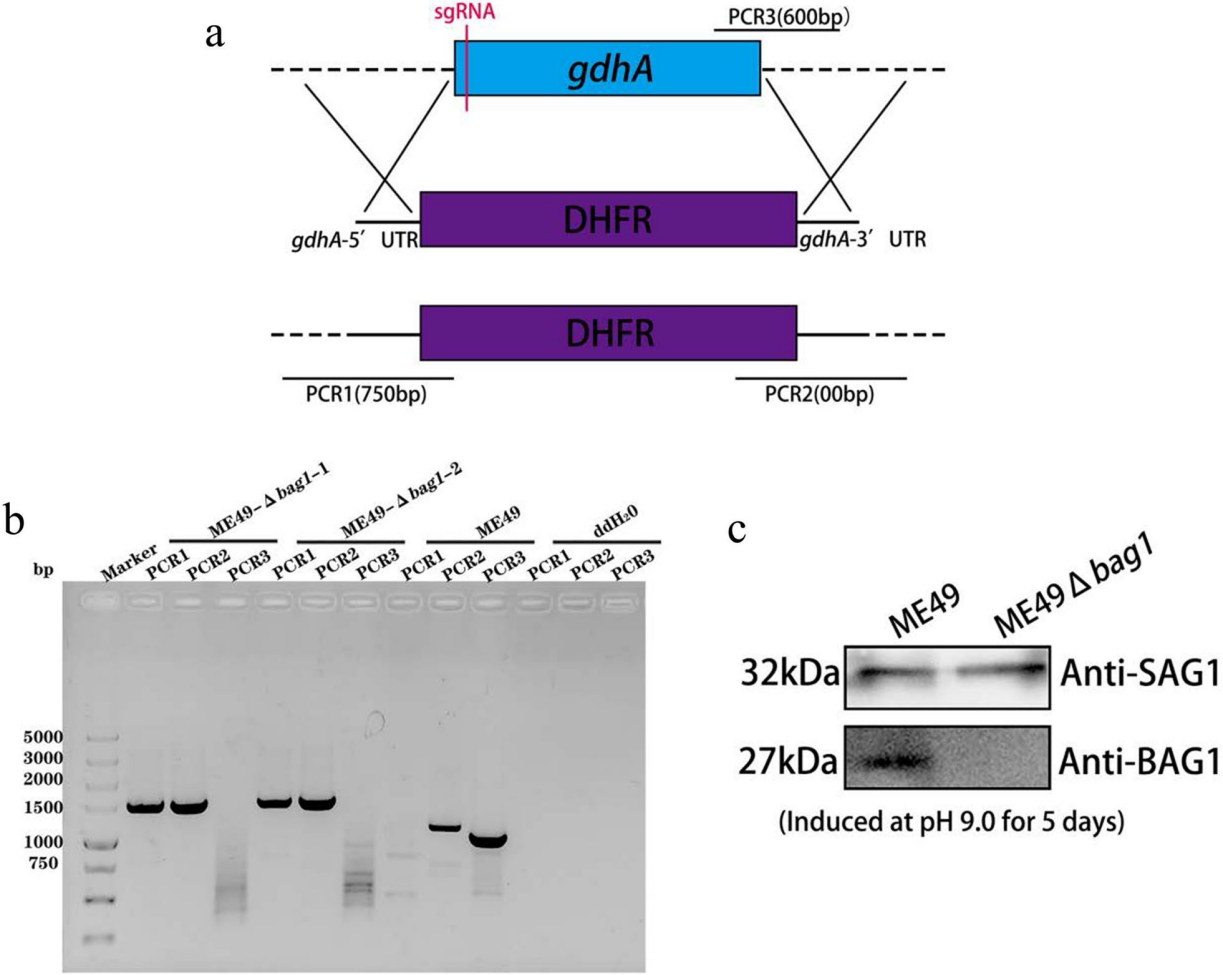
Construction and identification of *bag1* gene knockout ME49 strain. **a** Strategy for *bag1* gene knockout. **b**, **c** The recombinant ME49Δbag1 strain was harvested and lysed for DNA and protein extraction, and then identified by PCR(B), and western blot (**c**)

CRISPR/Cas9 technology was employed to knock out the *bag1* gene from the *T. gondii* ME49 genome (Fig. 1a). The CRISPR plasmid, which targeted the 5′-CDS of the *bag1* gene, along with the homologous template fragment of *bag1*-5′UTR-DHFR-*bag1*-3′UTR, was electrophoresed into ME49 tachyzoites. During the propagation of the tachyzoites, the coding region of the *bag1* gene was replaced with *bag1*-5′UTR-DHFR-*bag1*-3′UTR, and monoclonal recombinant strains were selected using pyrimethamine. Diagnostic PCR revealed positive results for PCR1 and PCR2 in the genomes of monoclonal knockout strains, whereas PCR3 yielded negative results. In contrast, PCR3 of the parental strain produced a positive result with the correct fragment size. While PCR2 amplification of the parental strain yielded a positive result, subsequent sequencing confirmed it to be the ME49 gene sequence, thus confirming the absence of *bag1* at the gene level (Fig. 1b). After 5 days of alkaline induction of ME49 and ME49Δ*bag1* parasite strains, tachyzoite proteins were extracted and used for western blot analysis using antibodies against SAG1 and BAG1. The absence of the BAG1 protein in the alkaline-induced whole proteins of the knockout strain serves as protein-level evidence that the *bag1* gene knockout was successful (Fig. 1c).

### The invasion and proliferation efficiency of ME49 in vitro and in vivo are not affected by *bag1* deletion

**Fig. 2:**
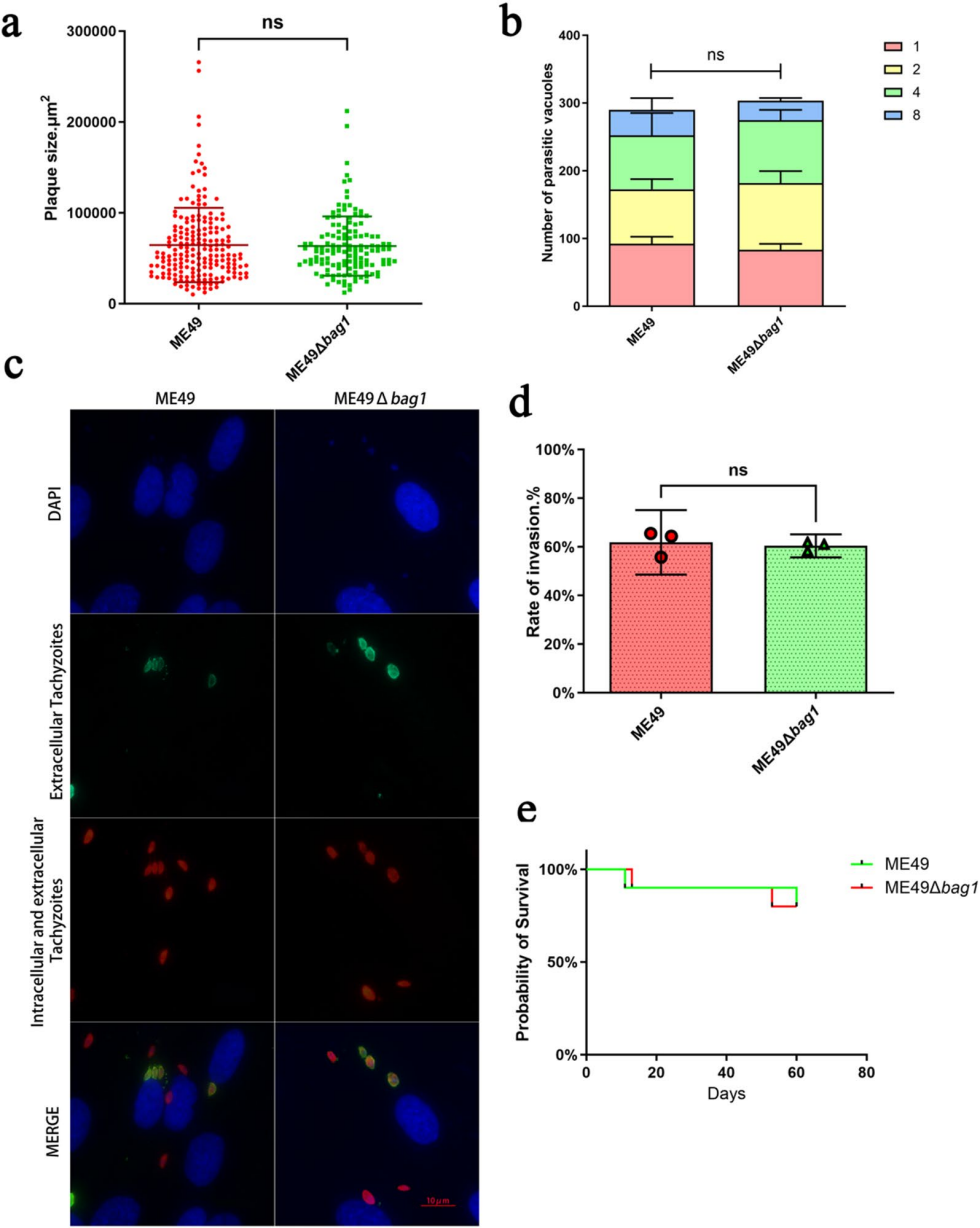
The invasion and proliferation efficiency of ME49 in vitro and in vivo is not affected by *bag1* deletion. **a** Plaque assay indicates no significant difference in plaque size between ME49 and ME49Δ*bag1* strains after infection in HFF cells (*P* > 0.05). **b** Intracellular replication assay indicates no significant difference in replication ability between ME49 and ME49Δ*bag1* (*P* > 0.05)*.* The numbers of parasitophorous vacuoles (PVs) containing 1, 2, 4, or 8 tachyzoites were calculated in 200 PVs and compared. **c**, **d** Invasion assay indicates no significant difference in the invasion efficiency between ME49 and ME49Δ*bag1* after infection in HFFs (*P* > 0.05). The typical immunofluorescence staining images indicate the extracellular tachyzoites labeled with red fluorescence and the intracellular tachyzoites labeled with green fluorescence (c). **e** The survival curves for the mice infected with ME49 and ME49Δ*bag1* strains for 60 days. The experiments were repeated three times for statistical analysis. The statistical significance between the data derived from ME49 and ME49Δ*bag1* was analyzed with *t*-test. n.s., not significant; *P < 0.05; **P < 0.01; ***P < 0.001, ****P < 0.0001

In our in vitro plaque assay, the plaques produced by ME49 and ME49Δ*bag1* were compared (Fig. 2a). Statistical analysis showed no significant difference in the plaque size between these two strains (*P* > 0.05). We further investigated the invasion efficiency and replication ability of ME49 and ME49Δ*bag1* using HFF cells. At 24 h post-infection, the number of tachyzoites in 200 PVs was calculated. The results indicated no significant difference in the proliferation rate between ME49Δ*bag1* and ME49 strains (*P* > 0.05) (Fig. 2b). At 1 h post-infection, the infected cells were fixed in paraformaldehyde, the extracellular tachyzoites were stained with green fluorescence, and the total tachyzoites were stained with red fluorescence (Fig. 2c). The parasite invasion efficiency was quantified by the ratio of intracellular tachyzoites to total tachyzoites, calculated within 100 HFF cells under a fluorescence microscope (Fig. 2d). The results demonstrated no significant difference in invasion efficiency between ME49 and ME49Δ*bag1* (*P* > 0.05) (Fig. 2d), suggesting that the disruption of the *bag1* gene did not impact the invasion capability of *T. gondii*.

To reveal whether *bag1* deletion would alter the virulence of the *T. gondii* ME49 strain, about 100 tachyzoites of ME49 or ME49Δ*bag1* were peritoneally injected into BALB/c mice. The survival of the mice was monitored daily for 60 days. As shown in Fig. 2e, no significant difference was observed in the survival curves of ME49 and ME49Δ*bag1,* indicating that *bag1* deletion would not affect the virulence of *T. gondii* in mice.

### The *bag1* gene facilitated in vitro bradyzoite differentiation and cyst formation in ME49

Given that BAG1 is exclusively expressed in bradyzoites, and that *bag1* knockout did not significantly change the capability of invasion, proliferation, and virulence of the ME49 strain, we subsequently investigated its role in bradyzoite differentiation through in vitro and in vivo studies.

**Fig. 3:**
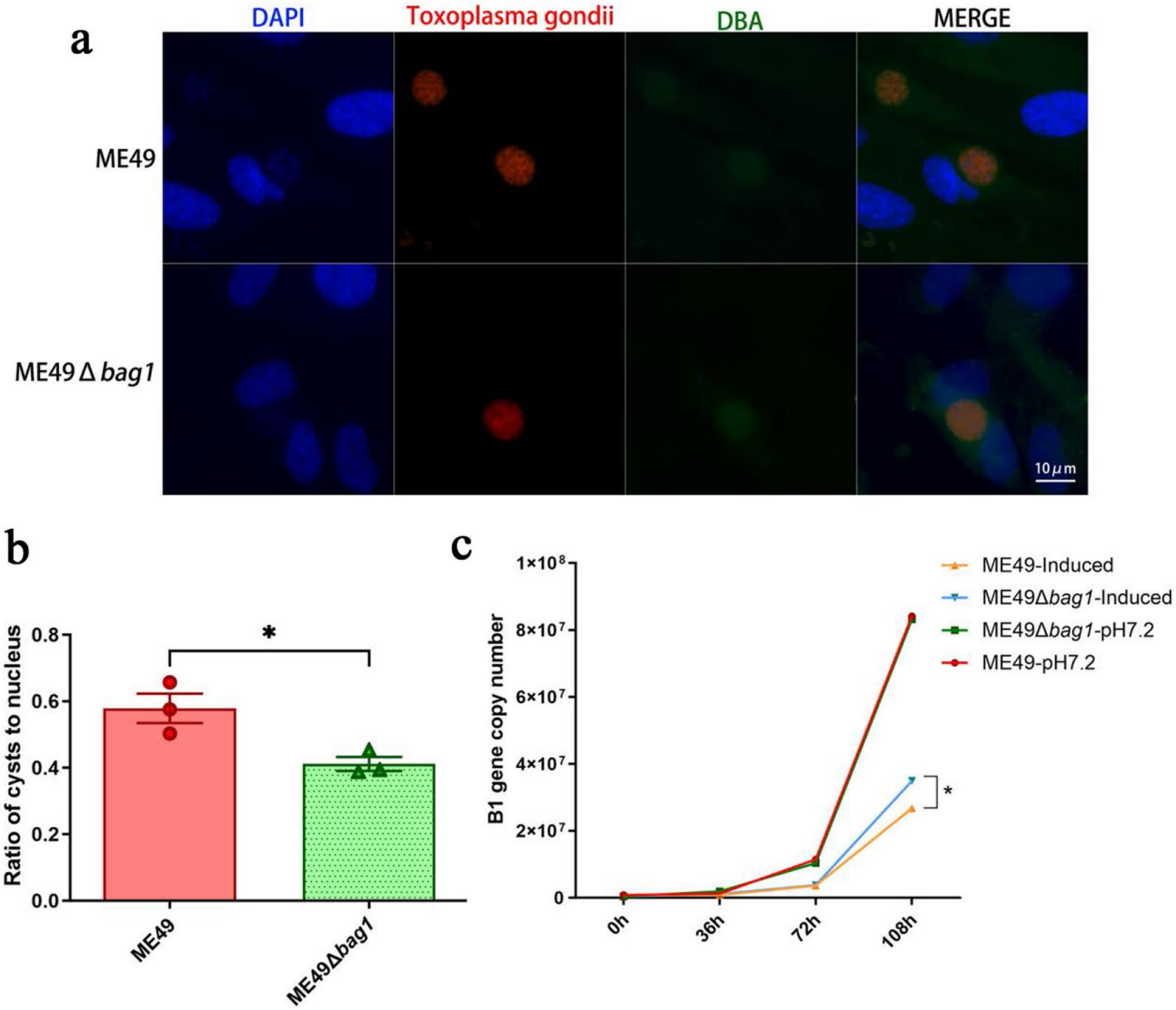
BAG1 facilitated bradyzoite differentiation and cyst formation in ME49. **a** Representative images depicting the differentiation in vitro of ME49 and ME49Δ*bag1* strains. Tachyzoites/bradyzoites were labeled with red fluorescence, using the primary antibody rabbit anti-*Toxoplasma* polyclonal antibody and the secondary antibody Alexa Fluor 594 goat anti-rabbit IgG, and the cyst wall was stained with green fluorescence using the FITC-conjugated *Dolichos biflorus* agglutinin (DBA). **b** The rate of in vitro cyst formation was calculated by dividing the number of DBA-positive cysts by the total count of 200 HFF cells. **c** *B1* gene copies were quantified by PCR using the genomic DNAs extracted from the ME49- and ME49Δ*bag1*-infected cells as the template. The culture condition and infected parasite strains are indicated. All images depict the results of three independent experiments. n.s., not significant; *P < 0.05; **P < 0.01; ***P < 0.001; ****P < 0.0001

HFF cells infected with ME49 and ME49Δ*bag1* were cultured in a pH 9.0 culture medium for 96 h to induce bradyzoite transformation. In vitro cysts were identified using the fluorescein isothiocyanate (FITC)-conjugated DBA under a fluorescence microscope (Fig. 3a). After counting the cysts in 200 HFF cells, it was observed that, compared with the ME49 strain, the ME49Δ*bag1* strain exhibited a significant reduction in cyst formation in vitro (Fig. 3b).

Genomes from the ME49 and ME49Δ*bag1* strains cultured in regular medium and alkaline conditions were collected. The parasite burden was assessed by detecting the *T. gondii* *B1* gene using qPCR. No significant difference was found in the parasite load between the ME49 and ME49Δ*bag1* strains when grown in a regular medium. However, under alkaline stress conditions, the parasite load was found to be higher in the ME49Δ*bag1* strain than the ME49 strain (*P* < 0.05) (Fig. 3c). This result indicated that the proliferation of the ME49Δ*bag1* strain was faster than that of the ME49 strain, possibly due to inhibited bradyzoite transformation in the ME49Δ*bag1* strain under in vitro alkaline stress conditions.

### The *bag1* gene promoted but is not essential for in vivo cyst formation of ME49

**Fig. 4:**
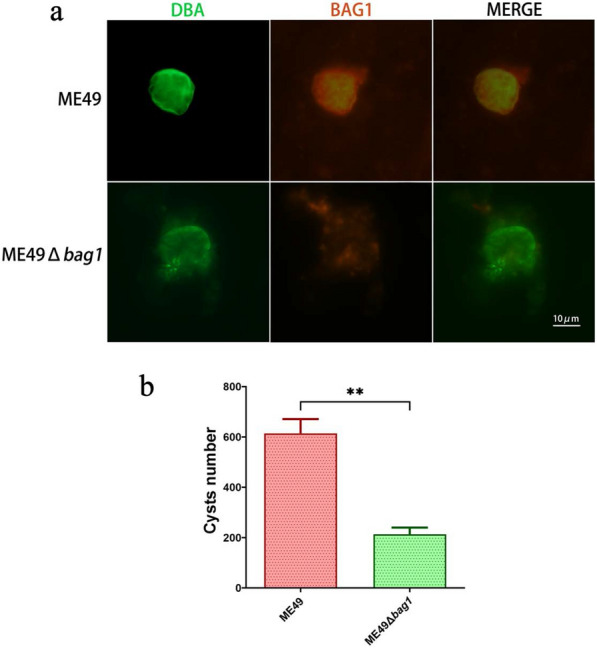
ME49 BAG1 facilitates but is not essential for in vivo cyst formation. **a** Representative images of fluorescence staining of brain cysts. **b** Quantitative assessment of the cyst load in the brains of mice at 60 days post-infection with 100 tachyzoites of ME49 and ME49Δ*bag1*. n.s., not significant; *P < 0.05; **P < 0.01; ***P < 0.001; ****P < 0.0001

Mice that remained alive at 60 dpi, as shown in Fig. 2e, were euthanized, and their brains were harvested and homogenized, and the cysts were counted following immunofluorescence staining (Fig. 4a). Although the ME49Δ*bag1* strain was still capable of forming cysts in mice, there was a marked decrease in cyst formation relative to the ME49 strain (Fig. 4b). This observation further underscored that the *bag1* gene is crucial for efficient cyst formation, but is not essential for cyst formation to occur.

### Detection of parasitic burden and immune responses in acute and chronic infection in a murine model

**Fig. 5:**
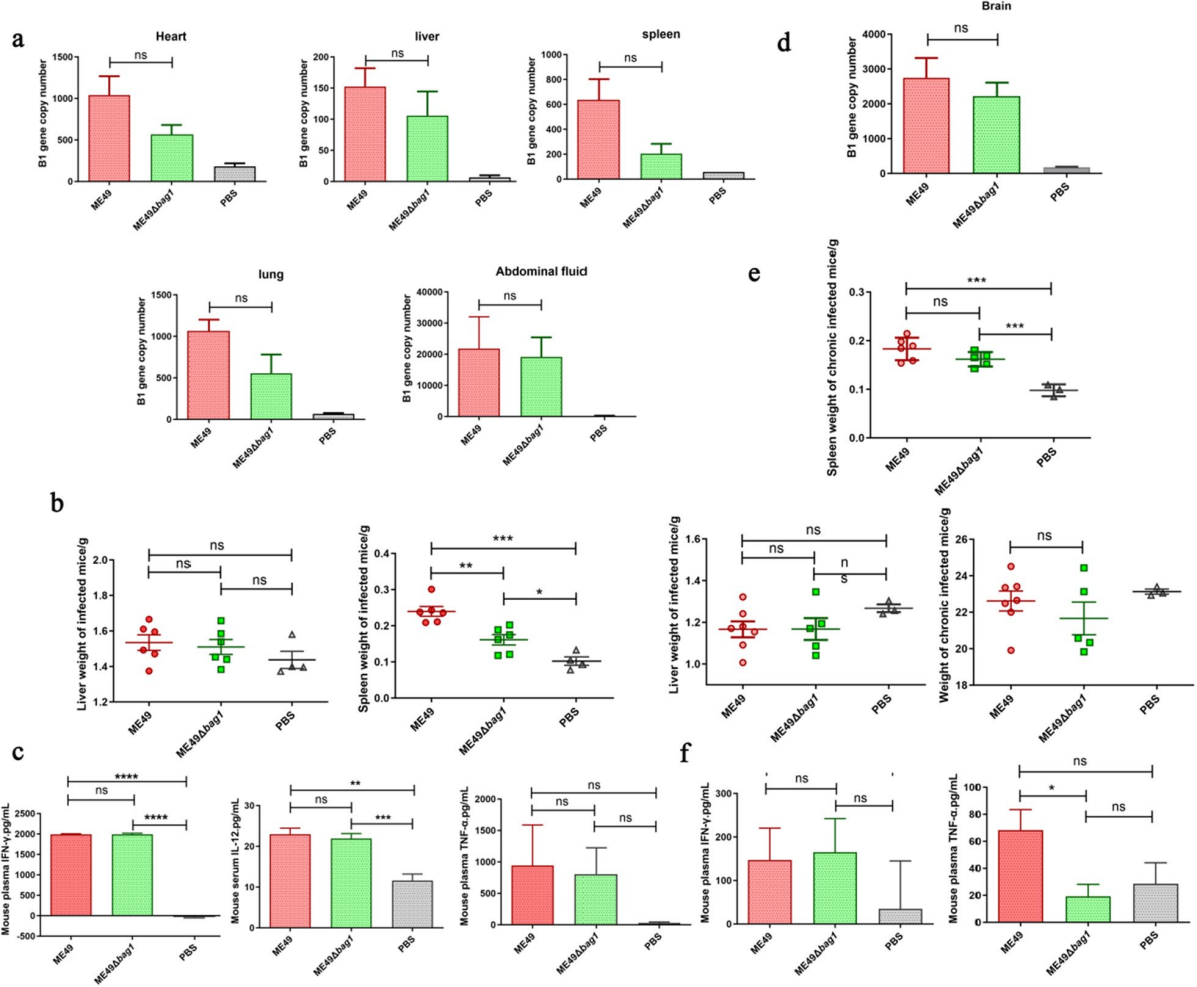
Comparison of the parasitic burden and immune responses between ME49 and ME49Δbag1 strain in acute and chronic infection. **a** Parasitic burden assessment in acutely infected mouse organs: 25 mg of heart, liver, and lung, and 10 mg of spleen collected from each mouse; for peritoneal fluid, 5 ml of PBS was used for lavage, from which 1 ml was taken. All these samples were subjected to DNA extraction and *B1* gene detection. **b** Liver and spleen weight measurement in mice with acute infection. **c** IFN-γ, IL-12, and TNF-α level detection in the serum of mice with acute infection. **d** Parasite load assessment in brain homogenates of mice with chronic infection; 100 μl of brain homogenate was taken from each mouse for DNA extraction and *B1* gene detection with qPCR. **e** Body, liver, and spleen weight measurement in mice with chronic infection. **f** IFN-γ and TNF-α level detection in the serum of mice with chronic infection. n.s., not significant; *P < 0.05; **P < 0.01; ***P < 0.001; ****P < 0.0001

We further investigated the underlying reason why the *bag1* gene is key to efficient cyst development but is not essential for cyst formation. About 1000 tachyzoites of ME49 or ME49Δ*bag1* strains were intraperitoneally injected into 5–6-week-old male BALB/c mice to establish an acute infection model. At 9 dpi, the mice were euthanized, and their hearts, livers, spleens, lungs, and peritoneal fluid were collected to assess the parasitic burden during acute infection. Furthermore, the brain homogenates of mice at 2 months post-infection (mpi) were examined for parasitic burden during the chronic infection. In the acute mouse infection model, no significant difference in the parasitic load was observed in the organs of infected mice (Fig. 5a) (*P* > 0.05). Similarly, no significant difference was observed in the brain parasitic load between the mice infected with ME49 and ME49Δ*bag1* strain in the chronic infection model, due to large intragroup variations (Fig. 5d) (*P* > 0.05).

We also compared the body weight of the mice at 2 mpi, and no significant difference was found (Fig. 5e, top panel) (*P* > 0.05). When continually comparing organ weights after infection, we found that the spleen weight in infected mice was significantly higher than that in uninfected mice. Specifically, spleen weight in mice acutely infected with the ME49Δ*bag1* strain was significantly lower than that in mice infected with ME49, suggesting that the immune response induced by the knockout strain during the acute phase might be weaker than that induced by the parental strain (Fig. 5b). However, this difference disappeared at 2 mpi (Fig. 5e, bottom panel). Furthermore, we conducted serum cytokine analysis in mice during acute and chronic infection periods (Fig. 5c and F). IL-12, which is involved in the initial differentiation of T cells into T helper 1 (Th1) cells and acts as a T-cell stimulatory factor, showed no significant difference in levels of IL-12 and INF-γ in mice serum during the acute phase, between the mice infected with ME49 and ME49Δ*bag1*. However, these cytokine levels in the serum of mice infected with either strain were significantly higher than those in uninfected mice, indicating a strong immune response and rapid disease progression. During the chronic phase, there was no significant difference in serum INF-γ levels between infected and uninfected mice, indicating a slow progression of disease and an immunological equilibrium at the chronic phase. In contrast, the serum TNF-α levels in ME49 and ME49Δ*bag1* infected mice were higher than those in uninfected mice, though the difference was not significant due to large inter- and intragroup variations. These results suggested that the immune response to *T. gondii* infection was reduced during chronic infection, and at 2 mpi, no significant impact on the immune response was observed in mice infected with either ME49 or ME49Δ*bag1*.

### Transcription detection of tachyzoite- or bradyzoite-specific genes important for cyst formation

Considering that the ME49Δ*bag1* strain yielded fewer cysts both in vitro and in vivo than the ME49 strain (Figs. 3b and 4b), while simultaneously eliciting a greater parasite burden in vitro (Fig. 3c), this may be due to the deletion of *bag1* leading to inhibited bradyzoite differentiation and promoted tachyzoite differentiation.

**Fig. 6:**
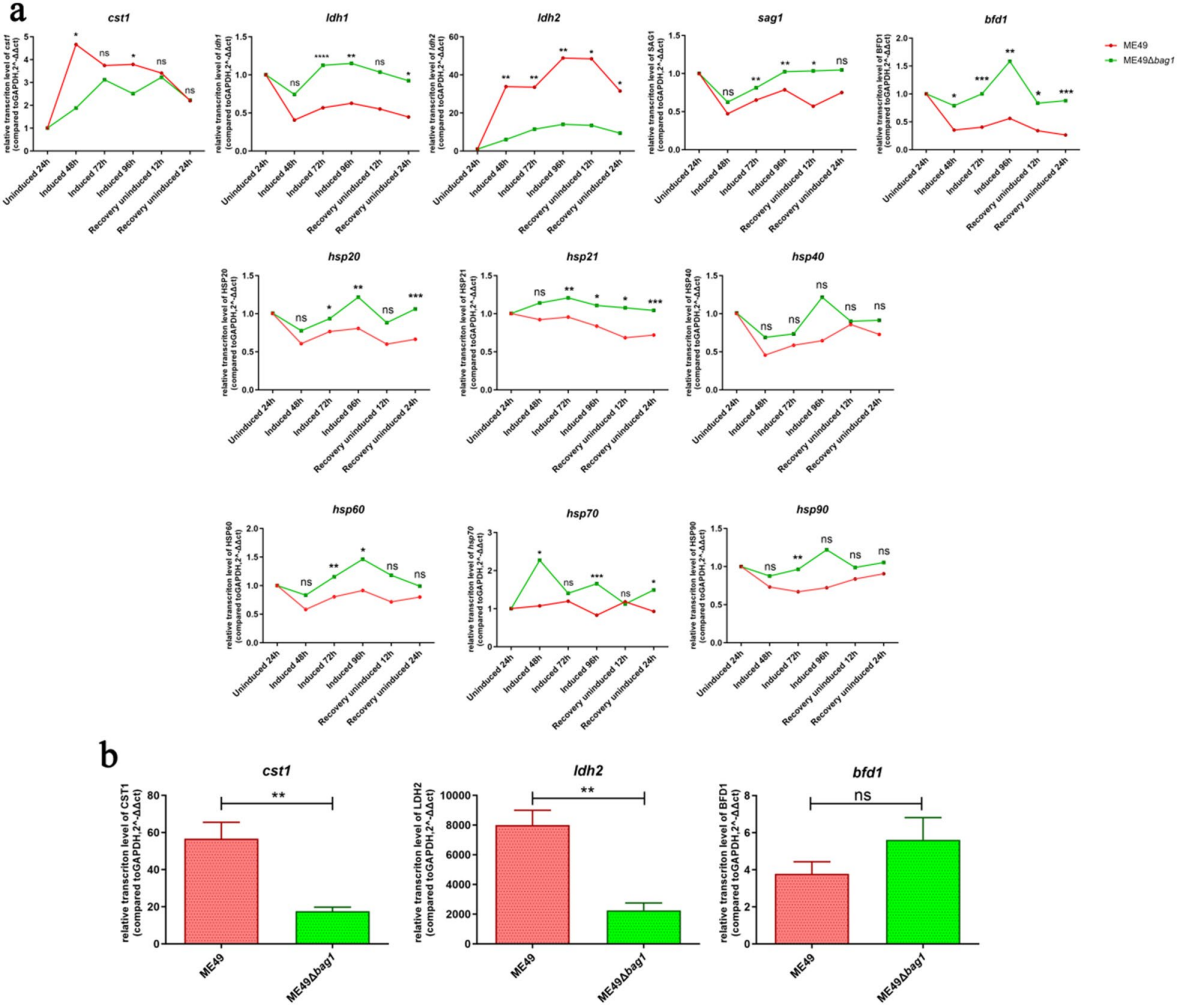
Transcription detection of the tachyzoite- or bradyzoite-specific gene that is important for cyst formation. **a** Gene transcription levels at various time points during the in vitro differentiation induction for ME49 and ME49Δ*bag1* strains. This figure illustrates the changes in transcription levels for *cst1*, *ldh1*, *ldh2*, *sag1*, *bfd1, hsp20, hsp21, hsp40, hsp60, hsp70*, and *hsp90* at different time points, including 24 h under standard medium, 48 h, 72 h, and 96 h under alkalized culture medium, and 12 h and 24 h of continuous culture following the removal of the alkalized culture medium. **b** Transcription levels of *cst1*, *ldh2*, and *bfd1* in brain homogenates of ME49 and ME49Δ*bag1* chronically infected mice. All images depict the results obtained from three independent experiments and have been plotted using mean values. n.s., not significant; *P < 0.05; **P < 0.01; ***P < 0.001; ****P < 0.0001

To verify this hypothesis, we infected HFF monolayer cells with an equal number of ME49Δ*bag1* and ME49 tachyzoites for 4 h, followed by in vitro alkaline induction. Subsequently, gene transcription levels were assessed at multiple time points. As illustrated in Fig. 6, throughout the alkaline induction, the ME49Δ*bag1* strain consistently demonstrated higher transcription levels of tachyzoite-specific genes, such as *sag1* and *ldh1*, when compared with the ME49 strain. The concurrent downregulation of bradyzoite-specific genes, such as *cst1* and *ldh2*, further supported this hypothesis.

Research has shown that the bradyzoite-formation deficient protein 1 (BFD1) binds near the transcription start sites of many genes upregulated during parasitic differentiation [5, 24]. The conditional expression of *bfd1* is sufficient to induce parasite differentiation in the absence of stress, identifying it as a key regulator of *T. gondii* bradyzoite formation. We observed that, compared with the parental strain, BFD1 protein remained upregulated during the early stages of differentiation in ME49Δ*bag1*, which may be an important reason why parasites can still differentiate into cysts after *bag1* knockout.

Upon examining gene transcription related to cyst formation in vitro, we further analyzed the gene transcription in the brain homogenates of mice chronically infected with the parasites (Fig. 6b). The results showed that the expression of the bradyzoite-specific genes *cst1* and *ldh2* was consistent with the in vitro experimental results, reaffirming the reduced number of cysts formed by chronic infection with ME49Δ*bag1* in the mouse brain. However, although the *bfd1* gene showed a slight upregulation in the brain homogenates of ME49Δ*bag1*-infected mice, the difference was not statistically significant (*P* > 0.05), possibly because the cysts had already formed and gradually stabilized.

### Compensatory upregulation of heat-shock proteins in response to decreased stress tolerance due to *bag1* deficiency

There are reports in the literature suggesting an interaction between BAG1 and HSP70, so we examined the change in transcription levels of *hsp70*. The HSP70 protein, a classic HSP induced in response to stress, and mainly expressed during the transition from tachyzoites to bradyzoites, plays an important role in the adaptation of *T. gondii *[24]. In our study, we observed that in ME49Δ*bag1*, the transcription trend of *hsp70* at various time points was opposite to that of the parental strain, with consistently higher levels of transcription. This may be due to a compensatory increase in *hsp70* under alkaline stress induced by the absence of *bag1*, a member of the sHSP. Therefore, we examined the transcription changes of other members of the HSP family during early differentiation induction in both strains. The results, as depicted in Fig. 6a, indicated that the knockout of the *bag1/hsp30* gene led to compensatory upregulation of HSPs HSP20, HSP21, HSP40, HSP60, HSP70, and HSP90, which further affected the expression levels of other members of the HSP family.

This suggested that complex interactions existed among different HSPs, which coordinated cell responses to environmental stress and significantly impacted the adaptability and survival of parasite strains. This finding elucidated the inhibition of *T. gondii* cyst formation in the absence of *bag1*, as the compensatory increase in HSPs can offset the compromised stress response, leading to the formation of a small number of cysts. It also further revealed the complex regulatory network of HSPs and other regulatory factors in parasite differentiation.

## Discussion

*Toxoplasma gondii* is a parasite that poses a huge threat to human health and the livestock industry. It is reported that the global seroprevalence of *T. gondii* antibodies is as high as one-third of the population [2], which underscores the importance of prevention and treatment. The interconversion between the tachyzoite and bradyzoite forms of *T. gondii* is the biological basis for its potential pathogenicity, and the mechanism of this conversion has been a focus of scientific research.

BAG1 is expressed in the cytoplasm of *T. gondii* bradyzoites and is identified as the sHSP HSP30. Its expression is regulated at the mRNA level [15] and is initiated early in the bradyzoite differentiation process, serving as a marker for bradyzoites [25]. Recombinant *T. gondii* BAG1 immunization has been proven to increase the survival rate and enhance protective immunity in mice, and reduce the formation of brain cysts [26]. The bradyzoite-specific antigen BAG1 can induce specific Th cell responses, and humoral reactions against bradyzoites and tissue cysts occur primarily in the early stages of infection [27]. Another study showed that immunization with pBAG1 resulted in a low IgG2a/IgG1 ratio, indicating that the BAG1 gene can induce a mixed Th1/Th2 type response [28]. Meanwhile, the T-cell response induced by BAG1 is associated with the production of IFN-γ, suggesting that BAG1 has potential as a vaccine candidate [27, 29]. The BAG1 gene is not essential for cyst formation or for the normal function of bradyzoites contained within tissue cysts [30, 31]. However, this does not imply that BAG1 has no role in bradyzoite differentiation, as it may participate in this process along with multiple genes that have redundant functions. Moreover, although BAG1 is not required for cyst formation, it influences the efficiency of cyst formation and can promote the formation of tissue cysts in the body [31].

In this study, we primarily investigated the phenotypic changes caused by the deletion of the HSP *hsp30/bag1* gene in the type II *T. gondii* strain ME49 and attempted to explain the reasons behind these changes. During the tachyzoite stage, the *bag1* knockout strain showed no significant differences in plaque formation, host cell invasion, replication, proliferation, or virulence in mice relative to the parental strain. This observation is consistent with the fact that the BAG1 protein is only expressed during the bradyzoite stage, and its absence does not affect the characteristics of the tachyzoite stage. However, upon inducing bradyzoite formation, the ME49Δ*bag1* strain demonstrated an accelerated early proliferation rate under alkaline stress, as indicated by the increased copy number of the *B1* gene. Nevertheless, the transcription levels suggested a slower rate of bradyzoite formation in the knockout strain. Therefore, we hypothesized that the phenomenon of accelerated early proliferation in the knockout strain under alkaline stress was attributable to the rapid division of tachyzoites that had not transformed into bradyzoites, rather than a slow proliferation of bradyzoites themselves.

Previous studies have indicated that BAG1 is not essential for cyst formation [8, 32]. Our study once again confirmed this point. After knocking out the *bag1* gene, parasites still formed cysts, but the number of cysts in the brains of mice was significantly reduced. In the mouse infection experiment, no difference was observed in parasite load in various organs between the knockout strain and parental strains. However, a significant difference was noted in spleen weight, suggesting a possible association between BAG1 protein and the immune system activation in mice. Upon *Toxoplasma* infection, the innate immune system detects the pathogen through pattern recognition receptors and induces proinflammatory cytokines such as TNF-α, IL-6, and IL-12, thereby activating effector cells to inhibit or kill the parasites [33]. TNF-α induces inflammatory proteins [34], IL-12 activates and stimulates the proliferation of NK cells, CD4^+^ T cells, and CD8^+^ T cells [35], while IFN-γ induces a cell-mediated immune response [36]. We measured the levels of cytokines in the serum of mice with acute and chronic infections and found that only TNF-α level showed a significant difference in the serum of mice 7 days after acute infection, indicating a reduced immune response to parasites in the knockout strain relative to the parental strain. In our study, we also investigated the changes in the transcription levels of some important genes related to cysts, including *cst1*, a cyst wall protein essential for maintaining cyst integrity. It is a mucin-like glycoprotein that provides the strength required for the parasite cysts and helps promote the persistence of bradyzoites [37, 38]. The transcription levels of *cst1* in the *bag1* knockout strain were consistently lower than in the parental strain, both in early in vitro induction and in mouse brain cysts, indicating a compromised cyst-forming ability.

Recent attention has been given to BFD1, a Myb-like transcription factor essential for the differentiation of *T. gondii* both in vivo and in vitro. BFD1 binds to the promoter of many stage-specific genes, accumulates during stress, and drives parasite differentiation, and it is considered a gene switch for *Toxoplasma* differentiation [5, 24]. With the discovery and attention to this gene, we further investigated the transcription level of *bfd1* during the differentiation process of the ME49Δ*bag1* strain. Compared with the parental strain, we observed a sustained upregulation of *bfd1* transcription in the ME49Δ*bag1* strain, which might be one of the factors that explain its continued ability to form cysts.

HSP70 is a HSP primarily expressed during the differentiation of tachyzoites into bradyzoites, playing a crucial role in the adaptive response of *T. gondii* to external stress [15–17]. HSP70 is also expressed during the transformation of bradyzoites to tachyzoites in *T. gondii*, and HSP70 plays an important role in the transformation of bradyzoites to tachyzoites during the reactivation of chronic toxoplasmosis [16]. It has been reported to interact with BAG1 [39]. Our study observed higher *hsp70* transcription levels in the ME49Δ*bag1* strain during differentiation, suggesting a reduced adaptability to external alkaline stress and a compensatory upregulation of *hsp70* in this knockout strain. Previous studies have categorized BAG1 as a sHSP family member, HSP30. Small HSP is a non-dependent molecular chaperone involved in the regulation of cellular differentiation [40], cell signaling, and apoptosis [41]. The interaction between HSP70 and HSP30 in *Neurospora crassa* suggests a co-chaperone relationship for denatured proteins [42]. Therefore, we concluded that the upregulation of HSP70 may also compensate for the absence of BAG1/HSP30. In the ME49Δ*bag1* strain based on this finding, we further examined the transcription levels of other HSP family members, including HSP40, HSP60, HSP90, HSP20, and HSP21, which were also upregulated in the ME49Δ*bag1* strain. HSP20 and HSP21 belong to the sHSP family and act as molecular chaperone proteins involved in processes such as protein folding, stability, and transport [43]. When cells are subjected to environmental stress or physiological damage, HSP20 can be activated and expressed to help the cells cope with these pressures. Similarly, HSP21, localized in the cytoplasm like HSP30/BAG1, is believed to compensate for some of the functions of HSP30 [44]. HSP40 typically interacts with other HSPs as a co-chaperone, participating in the regulation of protein folding, stability, and transport processes [13]. As a molecular chaperone, HSP60 participates in phase-specific induction of the *T. gondii* respiratory pathway [45–48]. HSP90 is a highly conserved molecular chaperone protein involved in crucial biological processes such as protein folding, stability, and transport [20]. HSP90 usually binds to adenosine triphosphate (ATP) and regulates the function and stability of target proteins through interaction with its client proteins. Similarly, another study showed that geldanamycin, a benzoquinone antibiotic, was able to bind and inactivate HSP90 function, blocking the interconversion of tachyzoites and bradyzoite, suggesting that this protein plays an important role in regulating phase interconversion [21]. These results all suggest that HSP90 is indispensable for the interconversion of *T. gondii* tachyzoites and bradyzoites. Additionally, HSP90 interacts with HSP70, which typically binds to client proteins in the early stages of protein folding and facilitates their correct folding, while HSP90 primarily participates in the later stages of protein stability and functional regulation [49].

In *T. gondii*, the complex interactions among HSPs significantly impact cellular growth and development. In our research, the knockout of the *bag1*/*hsp30* gene led to compensatory upregulation of HSPs, further affecting the expression of other HSP family members. These interactions coordinate the cellular response to environmental stress and significantly affect the parasite’s adaptability and survival.

This finding elucidates the inhibition of cyst formation in *T. gondii* due to the loss of *bag1*, and the compensatory upregulation of HSPs, which may help mitigate the impaired stress response and allow for the formation of some cysts. It also revealed the complex regulatory network of HSPs and other regulatory factors in parasitic differentiation. These insights not only deepen our understanding of *Toxoplasma* biology and pathogenic mechanisms but also open potential avenues for the development of new therapeutic strategies against *Toxoplasma* infection. HSPs play an important role in cellular stress response, and regulating their expression and function may provide a new approach for the treatment of *Toxoplasma* infection. For example, it has been shown that HSPs can assist pathogen survival and proliferation during infection [13–16, 20, 43, 50, 51], highlighting the importance of targeting compensatory upregulation of these proteins to address persistent parasite infection and cyst formation. By intervening in these compensatory upregulation mechanisms, it may be possible to diminish the parasite’s ability to survive within the host and form cysts, thereby mitigating chronic infection.

Future therapeutic developments may consider designing small-molecule inhibitors to specifically reduce the activity or expression of the aforementioned HSPs [52–54]. Such approaches could impair the ability of *Toxoplasma* to adapt to host environment changes, thereby reducing its chances of sustained survival and cyst formation in the host. However, successful implementation of this strategy requires a deeper understanding of the specific roles these HSPs play throughout the *Toxoplasma* life cycle and how they interact to promote cyst formation.

In addition, given the potential functional redundancy among members of the HSP family, the development of new therapies also needs to consider how to most effectively inhibit this compensatory response. Future studies should explore precise methods for regulating the expression or function of these proteins or targeting multiple HSPs simultaneously to achieve optimal therapeutic effects.

In summary, our study suggested that intervention in the compensatory upregulation of HSPs may provide promising avenues for controlling or treating *Toxoplasma* infection. While still theoretical, this finding is expected to guide future studies exploring new therapeutic strategies to combat infections and diseases caused by *Toxoplasma*.

## Conclusions

Our research indicated that in the absence of *bag1*, the diminished ability of *T. gondii* to respond to stress could be partially mitigated by the upregulation of other HSPs, leading to the formation of fewer cysts. This suggests a complex network of regulatory elements beyond BAG1 modulating the parasite’s interconversion between tachyzoites and bradyzoites, underscoring the critical compensatory roles played by HSPs in *T. gondii*’s life cycle adaptation strategies.

## Data Availability

All data in this article are publicly available. All data generated in this study are available from the corresponding author upon reasonable request.

## Abbreviations

HSP: Heat-shock protein
BAG1: Bradyzoite antigen 1
BFD1: Bradyzoite-formation deficient protein 1
SAG1: Surface antigen 1
*ldh1*: Lactate dehydrogenase 1
*ldh2*: Lactate dehydrogenase 2
*cst1*: Cyst wall protein 1
*gapdh*: Glyceraldehyde-3-phosphate dehydrogenase
DBA: *Dolichos biflorus* agglutinin
PVs: Parasitophorous vacuoles
BSA: Bovine serum albumin
FBS: Fetal bovine serum
MOI: Multiplication of infection
PVDF: Polyvinylidene difluoride
PMSF: Phenylmethylsulfonyl fluoride
RIPA: Radioimmunoprecipitation assay
HFF: Human foreskin fibroblast
SPF: Specific-pathogen-free
dpi: Days post-infection
HRP: Horseradish peroxidase
